# Cyclodextrin-Based Nanosponges: Overview and Opportunities

**DOI:** 10.3389/fchem.2022.859406

**Published:** 2022-03-24

**Authors:** Gianluca Utzeri, Pedro M. C. Matias, Dina Murtinho, Artur J. M. Valente

**Affiliations:** CQC, IMS, Department of Chemistry, University of Coimbra, Coimbra, Portugal

**Keywords:** cyclodextrins, nanosponges, crosslinked polymers, supramolecular interactions, responsive materials, multipurpose structures

## Abstract

Nanosponges are solid cross-linked polymeric nano-sized porous structures. This broad concept involves, among others, metal organic frameworks and hydrogels. The focus of this manuscript is on cyclodextrin-based nanosponges. Cyclodextrins are cyclic oligomers of glucose derived from starch. The combined external hydrophilicity with the internal hydrophobic surface constitute a unique “microenvironment”, that confers cyclodextrins the peculiar ability to form inclusion host‒guest complexes with many hydrophobic substances. These complexes may impart beneficial modifications of the properties of guest molecules such as solubility enhancement and stabilization of labile guests. These properties complemented with the possibility of using different crosslinkers and high polymeric surface, make these sponges highly suitable for a large range of applications. Despite that, in the last 2 decades, cyclodextrin-based nanosponges have been developed for pharmaceutical and biomedical applications, taking advantage of the nontoxicity of cyclodextrins towards humans. This paper provides a critical and timely compilation of the contributions involving cyclodextrins nanosponges for those areas, but also paves the way for other important applications, including water and soil remediation and catalysis.

## Graphical Abstract

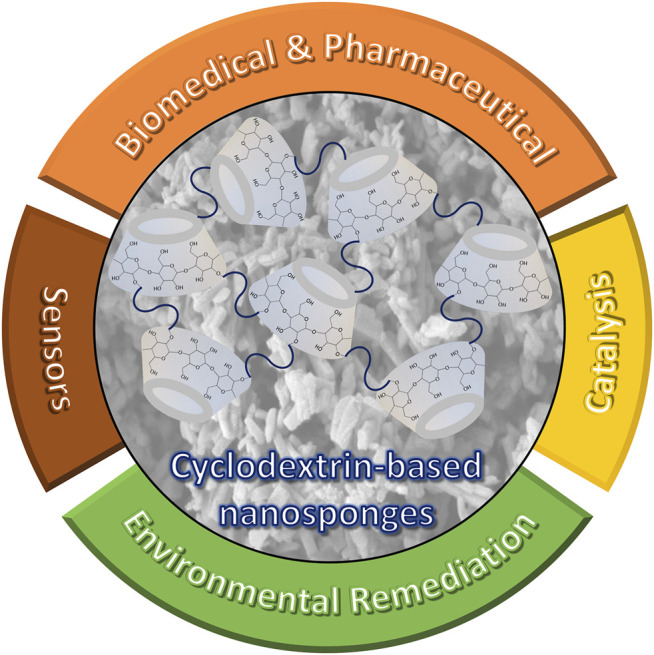

## Introduction

Cyclodextrins (CDs) are amazing molecules because of their peculiar and amphiphilic structure. They are natural oligosaccharides formed by α-(1,4)-linked glucopyranose units where the first three members are formed by 6, 7, or 8 glucopyranose units, labelled as α-, β-, or γ-cyclodextrin, respectively. Since the glucopyranose units are in the chair conformation (Figueiras et al., 2007; Messner et al., 2010), CDs have the form of a truncated cone or torus (Saenger et al., 1998), with a hydrophobic cavity. Due to their Lewis base character, they are able to form host−guest supramolecular structures (Schibilla et al., 2017; Neva et al., 2019; Usacheva et al., 2020; Wang J.-W. et al., 2021), and a hydrophilic outer surface as a consequence of hydroxyl groups positioned at both ends of the cavity (Saenger et al., 1998), allowing the interaction with polar compounds and, concomitantly, making them water soluble. The most varied compounds are those that can establish supramolecular interactions either through the hydrophobic effect or through, e.g., hydrogen bonds. To give some examples we can mention surfactants (Valente and Söderman, 2014; dos Santos Silva Araújo et al., 2021), drugs (Uekama et al., 1998; Rajbanshi et al., 2018; Saokham et al., 2018; Tian et al., 2020), organic pollutants (Cova T. F. G. G. et al., 2018; Filho et al., 2018; Liu Q. et al., 2020), dyes (Bezerra et al., 2020), and metal ions (Buvári and Barcza, 1989; Ribeiro et al., 2006; Prochowicz et al., 2017). The versatility of these oligosaccharides makes their application highly attractive in distinct fields such as pharmaceutical (the main one among them all) (Loftsson and Brewster, 1996; Loftsson and Duchene, 2007), food technology (Matencio et al., 2020c; Gonzalez Pereira et al., 2021), textile (Bezerra et al., 2020), wastewater treatment (Sikder et al., 2019; Chodankar et al., 2021; Cova et al., 2021), detergency (Schlüter et al., 2020) and paper (Aguado et al., 2019).

Cyclodextrins show high reactivity as a consequence of their hydroxyl groups able to suffer substitution or elimination. Due to these properties, cyclodextrins can be directly copolymerized with other monomers or grafted onto organic or inorganic materials (Mocanu et al., 2001; Cova T. F. et al., 2018, 2021; Gómez-Graña et al., 2021; Real et al., 2021) allowing a significant increase in the range of applications for CDs. Due to the high content in hydroxyl groups, cyclodextrins can easily form reticulated structures by co-polymerization with appropriate crosslinkers. Among the most used crosslinkers, epichlorohydrin (EPI) originates a hydrophilic gel with CD monomers connected by repeating glyceryl units. The polymerization with EPI occurs according to a well-known mechanism and the experimental conditions of synthesis are straightforward (Morin-Crini et al., 2018).

**FIGURE 1:**
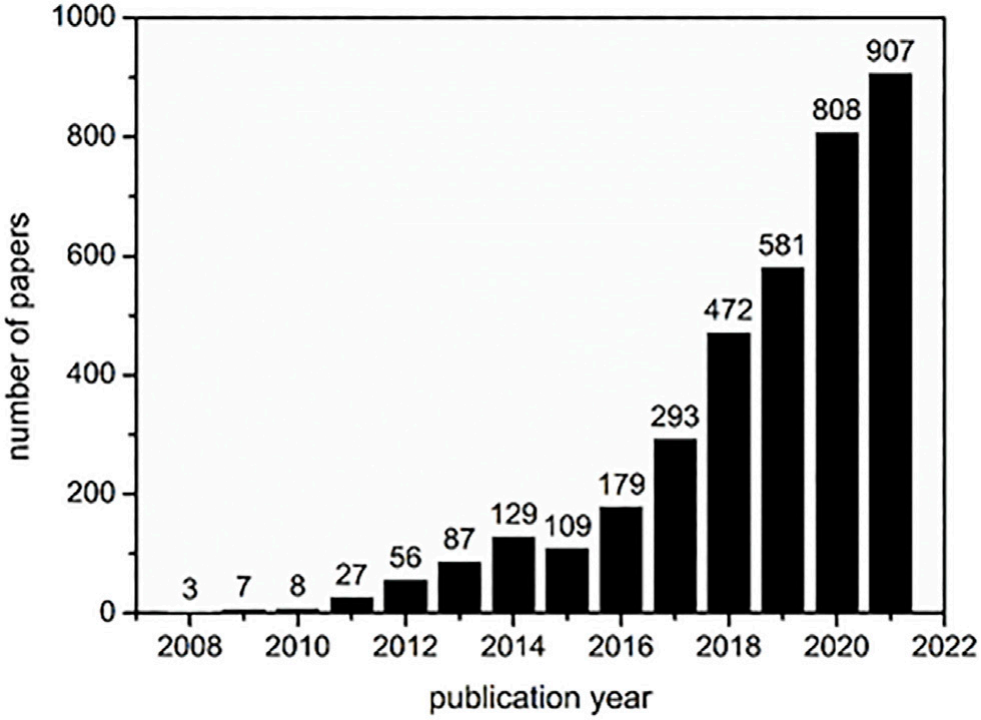
Number of papers that includes the keywords “cyclodextrin” and “nanosponge” cited by the Web of Science till 23.12.2021 (accessed at 12:41 GMT).

In recent decades a new type of crosslinked material, so called nanosponges (NSs) has emerged (Blasco-Tamarit et al., 2018; Trotta and Mele, 2019; Pawar and Shende, 2020a; Krabicová et al., 2020; Larin et al., 2020). Nanosponges can be defined as a hydrophilic, water-insoluble, supramolecular 3D‒hyper-reticulated nanoporous structures, showing a high stability over a wide range of temperatures and pH (Sherje et al., 2017). Thus, it is not surprising that cyclodextrins have also been used to synthesize these materials providing cooperative properties between the amphiphilicity and high surface area. However, it should be stressed that the application of cyclodextrin nanosponges (CDNSs) is still at an early stage. As can be seen from Figure 1, although the number of publications involving the keywords “cyclodextrin” and “nanosponge” is increasing exponentially, the absolute numbers are relatively low.

A meticulous analysis of data shows that the majority of published studies involving CDNSs is, by far, from the pharmaceutical area (Shrestha and Bhattacharya, 2020). The porosity, together with the amphiphilic properties of the cyclodextrin, permits the loading and solubilization of both hydrophilic and lipophilic molecules, allowing the increase in stability and bioavailability of drugs; on the other hand, the relatively low association constants characterizing the cyclodextrin-guest complexes (Houk et al., 2003; Kumar et al., 2020) [when compared with e.g., cucurbit(*n*)urils (Liu et al., 2005)] can be seen as an opportunity, since it enables not only the encapsulation (binding) but also the release and permeation of active substances (Mane et al., 2021; Pushpalatha et al., 2019). The same properties shown by CDNSs for an efficient drug encapsulation can also be used for the removal of toxic substances from the body, as is suggested in a few publications [e.g., (Varan et al., 2020)]. Other areas with identical relevance for the application of CDNSs are the removal of pollutants from wastewater (Kumari et al., 2020) and drinking water (Mhlanga et al., 2007) and heterogeneous catalysis (Jafari Nasab et al., 2018). Of course, given the versatility of these materials, their application in areas such as flame retardancy (Alongi et al., 2012), electrochemistry (Alidoost et al., 2022) and floriculture (at pre- and postharvest) (Seglie et al., 2012, 2013) can also be found.

**FIGURE 2:**
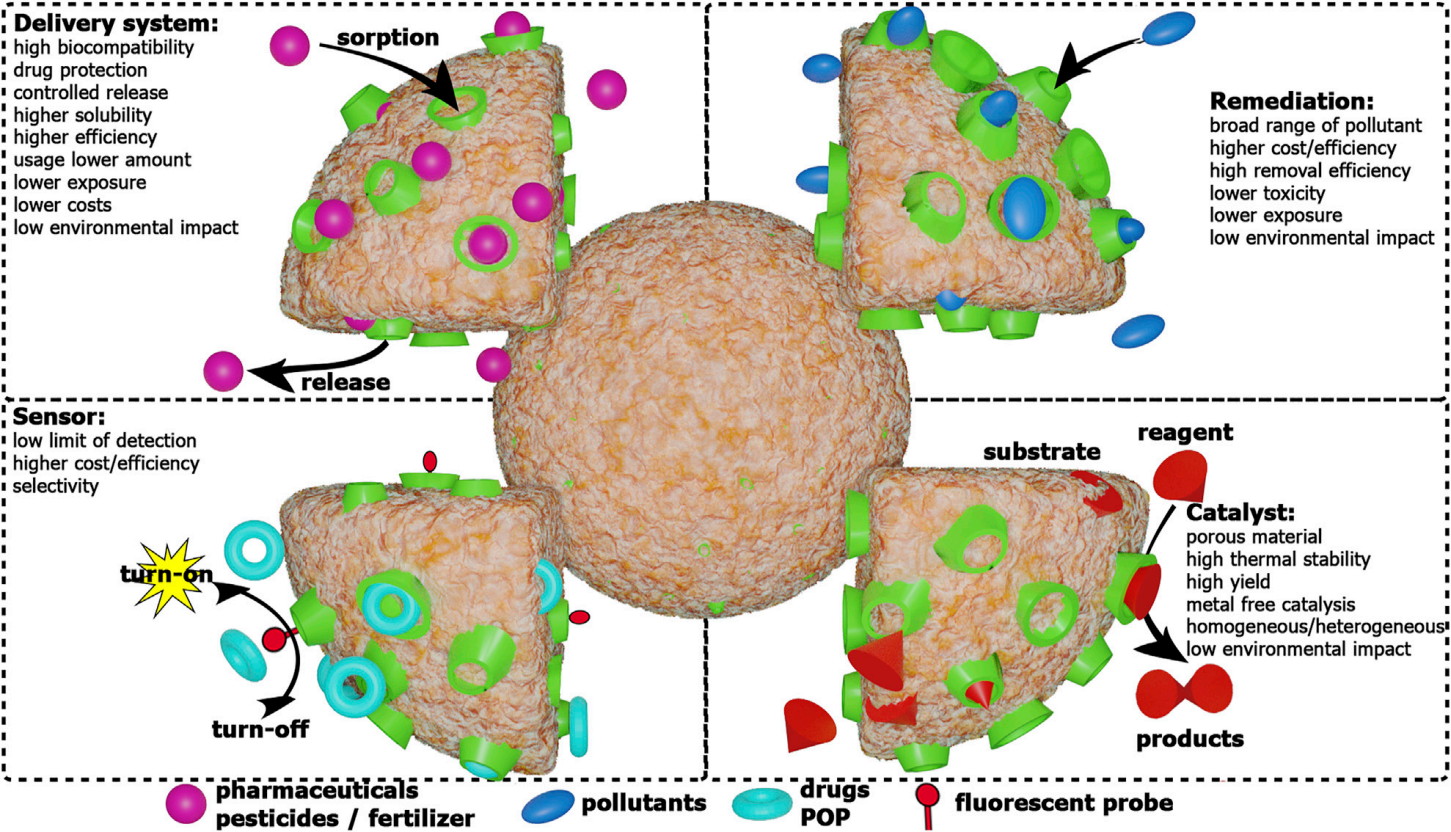
Schematic representation of CDNSs main applications and their advantages.

All these applications, as highlighted in Figure 2, will be discussed in detail in the following sections, along with the different paths and strategies for the synthesis of cyclodextrin-nanosponges. Finally, a critical assessment on the future perspectives for the application of these materials will be proposed.

## Cyclodextrin-Based Nanosponges: Synthesis and Properties

**FIGURE 3:**
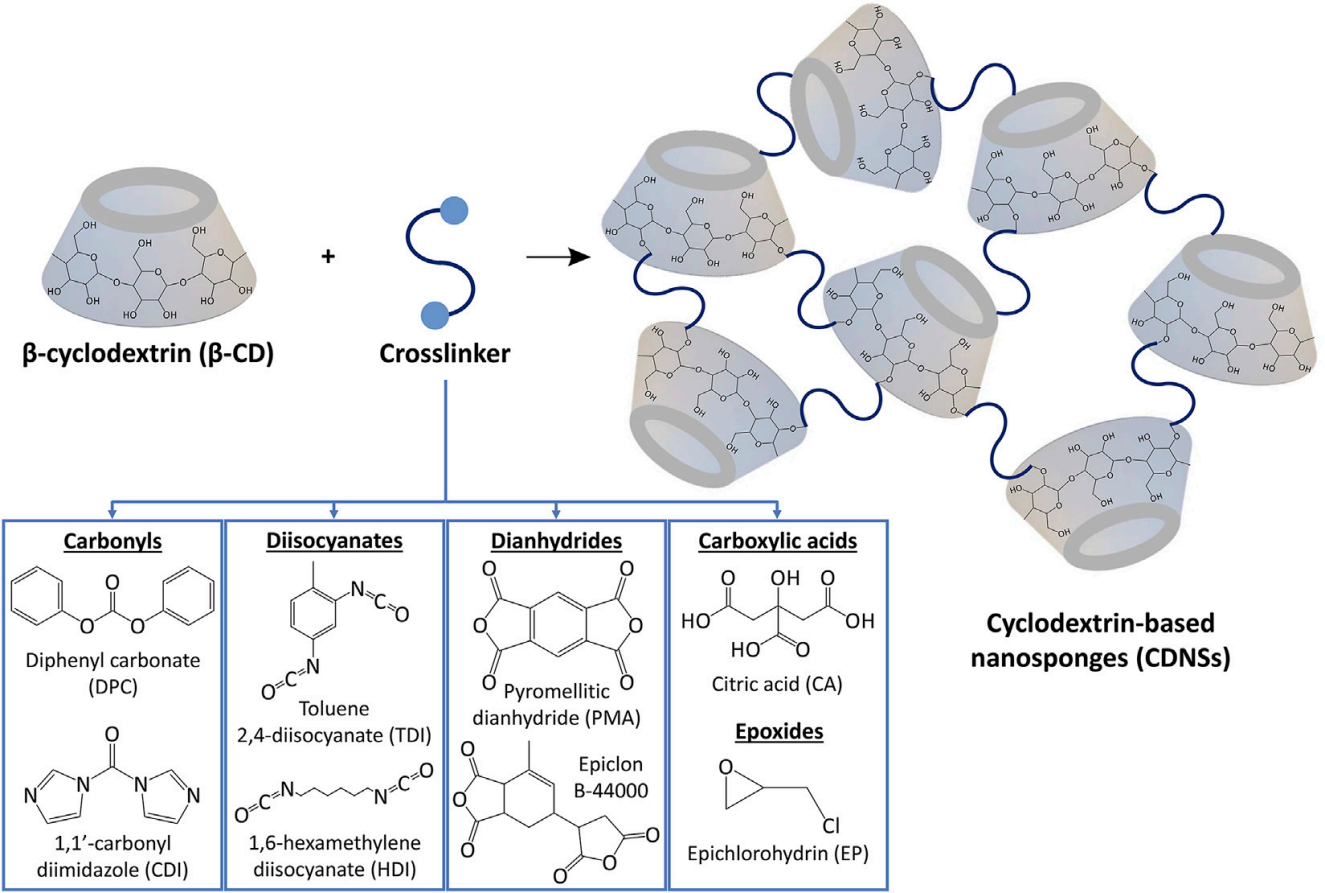
Synthesis of CDNSs through the reaction between β-cyclodextrin and a crosslinker, being carbonyls, diisocyanates, dianhydrides, carboxylic acids and epoxides the most common.

As a new family of spongy materials, CDNSs are solid, hyper-reticulated and nanoporous three-dimensional polymeric colloidal biodegradable nanostructures, typically having spherical shape (Cecone et al., 2020; Rizzi et al., 2021; Sadjadi and Koohestani, 2021a; Suvarna et al., 2021). In terms of structural regularity, CDNSs can be amorphous or crystalline frameworks (Singh et al., 2017). They can be directly obtained through the covalent binding between cyclodextrins and a multifunctional reactant (crosslinker)—see Table 1, which is possible thanks to the existence of reactive hydroxyl groups spatially located in the hydrophilic outer leaflet of CDs (Figure 3) (Morin-Crini et al., 2018; Gupta et al., 2021; Rubin Pedrazzo et al., 2022). Regarding CDs, both natural (αCD, βCD and γ CD) or chemically modified derivatives, such as 2-hydroxypropyl-βCD (Argenziano et al., 2019), sulfobutylether-βCD (Olteanu et al., 2014), carboxymethyl-βCD (Yakavets et al., 2020), tosylated-βCD (Sadjadi et al., 2019a) or halide-βCD (Utzeri et al., 2022) can be used. Nevertheless, βCD forms are preferred in the construction of CDNSs due to their cavity size, which confers greater stability, complexation ability and a larger number of encapsulation sites, as well as low cost and higher rates of production (Andaç et al., 2021; Guineo-Alvarado et al., 2021).

**TABLE 1:** Crosslinking agents used in the preparation of different categories of CDNSs.

CDNSs classes	Crosslinkers
Carbonate	Carbonyls: diphenyl carbonate (DPC); 1,1′-carbonyl diimidazole (CDI); dimethyl carbonate (DMC) and triphosgene
Carbamate	Diisocyanates: 1,6-hexamethylene diisocyanate (HDI); methylene diphenyl diisocyanate (MDI) Appell et al. (2018); toluene 2,4-diisocyanate (TDI) and toluene 2,6-diisocyanate Krabicová et al. (2020)
Ester	Dianhydrides: pyromellitic dianhydride (PMA); ethylenediaminetetraacetic acid dianhydride (EDTA) Ferro et al. (2017); Epiclon-B-4400 Gholibegloo et al. (2019a); dialcohol 2-hydroxyethyl disulfide (2-HEDS) can be use together with PMA to introduce disulfide bonds Trotta et al. (2016b)
	Carboxylic acids: citric acid (CA) Rubin Pedrazzo et al. (2019) and 2,6-naphthalene dicarboxylic acid (NDCA) Nait Bachir et al. (2019)
Ether	Epoxides: epichlorohydrin; 1,4-butanediol diglycidylether (BDE) Rizzi et al. (2021); E-51 epoxy resin Lai et al. (2012)
Polyamidoamine	2,2′-bis(acrylamido)acetic acid and its polyamidoamine derivates (PAA) formed by reaction with amines (such as 2-methylpiperazine) Swaminathan et al. (2010a)
Polyamine	Polyamines: 1,6-hexanediamine (am_6_), 1,8-octanediamine, 1,12-dodecanediamine (am_12_) Russo et al. (2016); Utzeri et al. (2022)
Other linkers	Dichloromethane Noothi et al. (2020) and *N*,*N*′-methylene bisacrylamide (MBA) Pawar et al. (2019)

### Synthetic Methods

Different crosslinkers and the crosslinking degree allow the construction of polymeric structures of distinct polarity and dimension, leading to macromolecules (CDNSs) with unique properties (Rubin Pedrazzo et al., 2022). The existence of lipophilic cavities in the building units (CDs) and the hydrophilic network in CDNSs, depending on the crosslinker’s nature, make these materials ideal candidates to increase the stability of sensitive and volatile compounds, as well as the solubility of lipophilic or lipophobic analytes (Asela et al., 2021; Guineo-Alvarado et al., 2021; Rezaei et al., 2021). Furthermore, the variety of synthetic methods used for the preparation of CDNSs also leads to polymers with distinct properties:

#### Hot Melting Procedure

This simple, reproducible, and solvent-free approach is based on the joint fusion of CD and typically a carbonyl linker, being diphenyl carbonate the most used. In general, the homogenization occurs at 90–130°C during at least 5 h, to ensure a complete crosslinking reaction (Sadjadi et al., 2019b; Iriventi et al., 2020). For further crosslinking, the mixture must be incubated for a longer period (Rezaei et al., 2021). At the end of the reaction a fine homogeneous powder is obtained (Jasim et al., 2020; Moin et al., 2020; Sadjadi and Koohestani, 2021a, 2021b; Srivastava et al., 2021). The powdered substance is repeatedly washed with water and/or acetone, and, generally, it is also subjected to Soxhlet extraction with ethanol or acetone, and even to an additional washing with sodium hydroxide (NaOH) solution (Kumar A. and Rao R., 2021; Sadjadi and Koohestani, 2021a, 2021b; Suvarna et al., 2021). Water allows the removal of CD in excess and ethanol/acetone allows the elimination of the unreacted crosslinker and still other impurities such as phenol or imidazole, formed when DPC or CDI linkers are used, respectively. Typically, the phenoxide ion formed from phenol is soluble in water, so the rinse with a base (NaOH) will ensure the total removal of this impurity, which can be observed using a ferric salt (Garrido et al., 2019; Kumar et al., 2021), UV-vis spectroscopy or HPLC (Sadjadi et al., 2018c).

#### Solvent Condensation Method

This strategy involves dissolving CD and a crosslinker in an appropriate solvent, namely, petroleum-based polar aprotic solvents such as DMF, DMSO, butanone or pyridine (Anandam and Selvamuthukumar, 2014), or green solvents to make the process more sustainable, such as water or aqueous solutions (Swaminathan et al., 2010a; Jafari Nasab et al., 2018) and deep natural eutectic solvents (NADES) (Cecone et al., 2020). If necessary, a catalyst can be added to reduce reaction time (Demasi et al., 2021). Typically, an excess of crosslinking agent is used in CD:crosslinker molar ratios in the range of 1:2 to 1:16 (Jain et al., 2020). In general, after the reaction, the recovery of NSs may involve precipitation using water, acetone (Khajeh Dangolani et al., 2019) or ethyl acetate (Cecone et al., 2018, 2019; Matencio et al., 2020b) among other possible solvents (Ferro et al., 2014; Appell et al., 2018; Garrido et al., 2019; Lo Meo et al., 2020; Pawar and Shende, 2021).

In the case of using dianhydride linkers alone or with 2-hydroxyethyl disulphide (Daga et al., 2016), the addition of triethylamine (Et_3_N) as basic catalyst is required, and the exothermic reaction occurs quickly at room temperature (Nazerdeylami et al., 2021; Suvarna et al., 2021; Yazdani et al., 2021). Using other linkers, such as HDI (Yazdani et al., 2021), MDI (Khajeh Dangolani et al., 2019), DMC and DPC (Singh et al., 2018), Et_3_N can also be introduced to increase the rate of the reaction, with or without an increase in temperature. Furthermore, the use of another base, 1,4-diazabicyclo(2,2,2)octane (DABCO), was reported to catalyse the reaction involving BDE as crosslinker in aqueous NaOH solution at 90°C or to catalyse the process using HDI and βCD at r. t. (Ramírez-Ambrosi et al., 2014). Basic conditions (NaOH) are also required for the reticulation of CD with EPI (Olteanu et al., 2014; Jafari Nasab et al., 2018; Liu X. et al., 2020). Other bases such as ammonia, pyridine and collidine can be used as catalysts as well (Trotta, 2011). According to the literature, when CA is chosen as linker, the reaction takes place under vacuum in water and at a temperature equal to or above its normal boiling point (Varan et al., 2020; Rubin Pedrazzo et al., 2022) or in NADES, such as a choline chloride/CA mixture, which acts both as solvent and co-reactant (Cecone et al., 2020). Sometimes, the addition of sodium hypophosphite monohydrate as catalyst is also required with this linker. Another example of this method involves the reaction between βCD and NDCA crosslinker, in aqueous media and using sulfuric acid as catalyst, to obtain the corresponding CDNSs after 2 days at 100°C (Nait Bachir et al., 2019).

#### Interfacial Condensation Method

This method involves the complete dissolution of the CD in an alkaline aqueous phase, pH > 10, and the crosslinking agent in an organic one (methylene chloride, butanone or chloroform) (Shende P. et al., 2015; Desai and Shende, 2021).

#### Emulsion Solvent Diffusion Method

Based on the phenomenon of emulsification, this process consists of two immiscible phases: one internal and one external. The internal phase is formed when the crosslinker is added dropwise, under constant magnetic stirring, to a solution containing CD and an inclusion analyte in a polar aprotic solvent (usually DMF). The external phase is an aqueous solution to which the internal phase is then added drop by drop, under vigorous stirring, at room temperature. The suspension obtained is lyophilized and the CDNSs are subsequently dried (Gangadharappa et al., 2017).

#### Ultrasound-Assisted Synthesis

Through the application of ultrasonic vibration, it is possible to promote crosslinking of CD with an appropriate linker, in a certain molar ratio, and in the absence of solvents, thus constituting an environmentally friendly process. Spherical uniform size particles are formed (Ciesielska et al., 2020; Jain et al., 2020; Omar et al., 2020). The sonication is useful either for the melting or for the solvent condensation method (Swaminathan et al., 2013).

#### Microwave-Assisted Synthesis

Conventional and ultrasound heating methods lead to non-uniform transformations due to the occurrence of thermal gradients and, consequently, to longer reaction times and scalability problems. As such, the promotion of reactions by microwave irradiation makes them four times faster than the melting approach (Andaç et al., 2021), and more reproducible and scalable due to uniform and controlled heating provided by microwave irradiation. Thus, using microwave synthesis, it is possible to obtain highly crystalline CDNSs with a narrow particle size distribution (Ciesielska et al., 2020) by reacting CD with an appropriate crosslinker (mostly DPC), using polar aprotic solvents such as DMF (Anandam and Selvamuthukumar, 2014; Zainuddin et al., 2017; Sharma et al., 2021). As the solvent condensation synthesis can be performed using microwave irradiation, Vasconcelos et al. (Vasconcelos et al., 2016) reported the use of tin octanoate catalyst to promote the reaction between βCD and HDI crosslinker using DMF as solvent in a microwave system at 80°C for 30 min.

#### Mechanochemical Synthesis

CD-crosslinker reaction can also be induced by mechanochemistry (Jicsinszky and Cravotto, 2021), the direct absorption of mechanical energy, capable of activating chemical bonds. Usually, this type of activation occurs between solids or solidified reactants in ball mills, in the absence of solvents or minimizing their use as much as possible, contrary to what happens conventionally, whereby a large quantity of solvents, mostly derived from fossil fuels, are used. So, mechanosynthesis is a more sustainable method, where mass transport and energy dispersion are guaranteed through efficient grinding in the solid state. Although solvents such as acetone and ethanol continue to be used in CDNSs’ purification, they are relatively volatile, in contrast with high boiling point polar aprotic solvents (DMSO or DMF), that have complex recycling processes. The use of ball mills has disadvantages such as difficult scalability, the impossibility of precise temperature control (although it does not exceed 72°C in CDNSs’ synthesis), and the use of closed containers that increase the polycondensation reaction time due to the impossibility of water removal during these batch processes. These drawbacks can be overcome by using twin-screw extruder reactors, which allow not only a tight temperature control but also makes processes more scalable by transitioning from a batch approach to a continuous process. In general, mechanosynthesis is a simple, economical, and faster strategy for obtaining CDNSs. Two examples are: 1) the use of ball mills to obtain CDNSs using CDI as cross-linker in 3 h; and 2) the use of said extruder for the preparation of CDNSs using βCD, CA crosslinker, and sodium hypophosphite monohydrate as catalyst. In the latter case, the equipment is preheated to a temperature between 120 and 180°C and the solid blend is slowly inserted into it and the process lasts between 5 and 25 min, contrary to the conventional approach that takes place in vacuum for at least 4 h, using water as solvent (Rubin Pedrazzo et al., 2020, 2022; Trotta and Pedrazzo, 2020).

#### Chain-Growth Polycondensation Method

The conventional step growth procedures are based on a polycondensation reaction in which monomers react with each other and, subsequently, a new monomer reacts with the polymer under construction, presenting both functional groups with identical reactivity. However, it is possible to evolve to a chain-growth polycondensation method, in the case of the reactive group at the polymer chain’s end, formed by reaction with the monomer, is stable and more reactive than the monomer itself (Yokozawa and Ohta, 2016). Thus, an initiator can be used to promote polymer growth by creating points of high reactivity at its tip, so that the binding of a new monomer readily occurs, and so on, in these reactive ends, allowing the increase of the chain. CDNSs with reduced polydispersity are obtained, as the monomers do not interact with each other, but selectively connect with the reactive terminals. A chain-growth approach reported by Khalid et *al*. (Khalid et al., 2021) is based on the use of βCD together with acrylic acid monomer in the presence of ammonium persulfate as initiator and MBA as crosslinking agent.

### Properties

CDNSs are colloidal systems with an average diameter size generally below 1 µm and with a narrow size distribution, denoted by a polydispersity index (PDI) less than 0.7, which is typical of monodispersed particles (Kumar et al., 2021). Their high ζ-potential (±30 mV), mostly showing a negatively charged surface, means that the particles easily disperse in water to form stable suspensions, whereby they tend to repel electrostatically, not acting as surfactants, as they do not aggregate (Trotta et al., 2012; Suvarna et al., 2021). However, NSs can swell upon water absorption appearing to have a gel structure, as hydrogels. Thus, although swelling is not directly related to uptake capacity in CDNSs, PMA and CA CDNSs have a greater degree of swelling at small quantities of crosslinker compared to carbonate and carbamate ones, decreasing with the increase of crosslinker:CD ratio, because of strong crosslinking formation and loss of structural elasticity (Trotta, 2011; Hoti et al., 2021; Rubin Pedrazzo et al., 2022). Polyamidoamine CDNSs also have a huge swelling in aqueous media and they bear both acidic and basic groups (Swaminathan et al., 2010a). In addition to crosslinking, there may also be some branching with PMA and CA linkers, culminating in the formation of carboxylic acid groups, which can cause ζ-potential oscillations, as they are pH-sensitive, giving them the possibility to host cations and organic molecules simultaneously (Varan et al., 2020). The use of EPI linker leads to more hydrophilic NSs than those obtained with carbonyl, dianhydride or diisocyanate linkers.

Characteristics of nanosponges’ dimension, surface area (S_BET_), porous network, charge, and ζ-potential are determinant factors for the interaction with an analyte. These properties (in Table 2) are greatly affected by CDNS structure, which depends on the linker and CD form chosen, CD:crosslinker proportion, solvent, catalyst and synthetic conditions. All parameters play an important role on the properties of CDNSs in terms of loading capacity and efficiency (in general, higher in crystalline CDNSs and in native CDs) (Osmani et al., 2018; Sharma et al., 2021), release profile and solubility, being cross-linked cyclodextrin polymers often insoluble in water and organic solvents (Sherje et al., 2017; Venuti et al., 2017).

**TABLE 2:** Comparative properties of different types of CDNSs.

Nanosponge		Method	Structure	Mean size/nm	PDI	ζ-Potential/mV	S_BET_/m^2^ g^−1^	Pore diameter/nm	Pore volume/cm^3^g^−1^
βCD:DPC Kumar et al. (2021); Liu et al. (2020b); Sharma et al. (2021); Sadjadi et al. (2018a); Sadjadi et al. (2017b); Rao et al. (2013)		Melt	C/A	<664	<0.45	− (3–22)	9.7 (1:2)	∼ 11.1 (1:2)	∼ 0.03 (1:2)
βCD:DPC Shringirishi et al. (2017); Pushpalatha et al. (2018b); Singireddy et al. (2016); Liu et al. (2020b); Gupta et al. (2021)		Solvent	A	135–500	<0.43	± (12–35)	2.2 (1:2)	13.3 (1:2)	0.0075 (1:2)
βCD:DPC 1:4 Guineo-Alvarado et al. (2021); Singireddy et al. (2016)		MW	C	153 ± 8	0.11 ± 0.01	28 ± 2	<2.0	—	—
βCD:CDI 1:4 Rao et al. (2018); Sabzi and Kiasat (2018); Kardooni et al. (2020); Desai and Shende (2021)		Solvent	A	473 ± 1	0.24 ± 0.06	−(39 ± 1)	10.9	4.86	0.013
		Interfacial	—	173 ± 1	0.22 ± 0.03	−(33 ± 1)	—	—	—
CD:HDI Salgın et al. (2017); Mhlanga et al. (2007)		Solvent	A	420×10^3^	—	—	1.7–3*.*5	—	0*.*01
CD:TDI Mhlanga et al. (2007); Deshmukh and Shende (2018); Varan et al. (2020)		Solvent	A	367 ± 2	0.25 ± 0.02	−(26 ± 2)	1.7–3*.*5	—	0*.*01
αCD:MDI 1:10 Khajeh Dangolani et al. (2019)		Solvent	A	100–200	—	—	11.9	35.9	0.11
βCD:PMA Gabr et al. (2018); Rao et al. (2018); Pushpalatha et al. (2018b)	1:4	Solvent	A	605 ± 18	0.31 ± 0.03	−(61 ± 2)	1.21	120.3	0.04
	1:6			264 ± 16	0.26 ± 0.01	−(60 ± 2)	0.573	484.8	0.07
	1:8			477 ± 23	0.72 ± 0.06	−(60 ± 3)	0.393	299.9	0.03
GSH-NS: βCD:PMA/2-HEDS Palminteri et al. (2021); Daga et al. (2020)		Solvent	A	203 ± 14	0.20 ± 0.01	−(32 ± 3)	—	—	—
βCD: Epiclon Gholibegloo et al. (2019b)	1:2	Solvent	A	300 ± 10	0.27	−(24 ± 2)	0.4	105	-
	1:4			206 ± 9	0.20	−(27 ± 2)	6.0	22	
	1:8			140 ± 6	0.19	−(37 ± 2)	9.3	16	
βCD:CA Cecone et al. (2020)		NADES	A	<20×10^3^	—	5–18	9.0	—	—
βCD:CA 1:8 Rubin Pedrazzo et al. (2022)		Solvent	—	800	—	−(29 ± 11)	1.30	—	—
		Extrusion	—		—	−(26 ± 7)	0.93	—	—
βCD:EPI Jafari Nasab et al. (2018); Liu et al. (2020b)		Solvent	A	167 ± 8 (1:8)	0.58 (1:8)	−(37 ± 2) (1:8)	10.9 (1:10)	4.86 (1:10)	0.014 (1:10)
CD:am_6_ Utzeri et al. (2022); Russo et al. (2016)		Solvent	A	446 ± 30	0.38 ± 0.05	41 ± 6	21.8	24.3	0.13
CD:am_12_ Utzeri et al. (2022); Russo et al. (2016)		Solvent	A	438 ± 38	0.43 ± 0.03	54 ± 7	17.9	7.5	0.03
PAA:NS Swaminathan et al. (2010a)		Solvent	—	410–502	0.10–0.12	−(32–35)	—	—	—
βCD:acrylic acid Khalid et al. (2021)		Chain-growth	A	275 ± 29	0.28	−(41 ± 5)	—	—	—

A: amorphous; C: crystalline.

The amount of crosslinker influences the surface area and porosity. Normally, with the increase in its proportion in relation to CD, the smaller the pore diameter will be, and a higher degree of crosslinking will lead to a greater S_BET_ value, which can create a superior polymeric interconnection forming a material with greater porosity. This situation is denoted in βCD:Epiclon NSs (Table 2), in which the degree of crosslinking increases from 61 to 94% with the increase of crosslinker amount (from 1:2 to 1:8 M ratios) (Gholibegloo et al., 2019b).

The NSs are thermally stable structures up to 300°C and resistant to organic solvents, and their formation can be screened by infrared spectroscopy, through bands that do not exist in the original CDs. For example, carbonate CDNSs present C=O elongation of the carbonate linkage (Darandale and Vavia, 2013) (Singireddy et al., 2016) at 1720–1780 cm^−1^; carbamate CDNSs show characteristic peaks at 1700, 1630 (due to amide-I-like carbonyl stretching) and 1550 cm^−1^ (assignable to amide-II-like N−H bending) (Lo Meo et al., 2020), and the absence of the N=C=O vibration band at 2270 cm^−1^ (Leudjo Taka et al., 2020); ester CDNSs have a band at 1720–1735 cm^−1^ corresponding to the C=O ester bonds (Gholibegloo et al., 2019a; Varan et al., 2020).

As tiny mesh-like structures, CDNSs may encapsulate many molecules, making them useful in solubility, cytotoxicity and bioavailability enhancement, drug delivery, protection and transport of unstable molecules, catalysis, environmental remediation, chemical and biological sensing (important in diseases diagnosis), gas carrying and in release of enzymes, proteins, vaccines, and antibodies, as a form of treatment. Their safe, biodegradable, non-toxic and biocompatible nature make them promising materials for the aforementioned applications.

Compared with activated carbon (with S_BET_ = 600–700 m^2^ g^−1^), CDNSs have lower surface area but similar interaction capacities for lipophilic molecules, which means that these guests are both adsorbed on the surface and carried into the bulk of the NSs during inclusion complex formation or internal diffusion, leading to higher apparent stability constants compared to those using free CDs (non-bond) ( ≤ 10^4^ M^−1^), which do not have the ability of host hydrophilic or high molar mass analytes. Despite the formation of inclusion compounds being highly favoured in water, it is reversible in organic solvents (such as ethanol), which makes CDNSs’ full recover and reuse easy, without requiring hazardous burning techniques for partial regeneration, as is the case with activated carbon (Trotta, 2011).

## Applications

### Pharmaceutical and Biomedical Applications

Cyclodextrin-based nanosponges have shown a great potential for applicability in pharmaceuticals and have been widely explored for this purpose in the last decade. (Caldera et al., 2017; Sherje et al., 2017; Krabicová et al., 2020). One of the most relevant applications is in drug delivery, highlighting in this field the use of CDNSs for cancer therapy (Subramanian et al., 2012; Trotta et al., 2012, 2014; Lakkakula and Krause, 2013; Swaminathan et al., 2016; Venuti et al., 2017; Lembo et al., 2018; Osmani et al., 2018; Allahyari et al., 2019; Kumar and Rao, 2019; Menezes et al., 2019). This topic has been the subject of numerous review articles and book chapters, some of which are quite recent (Ciesielska et al., 2020; Jain et al., 2020; Tannous et al., 2020, 2021; Andaç et al., 2021; Babadi et al., 2021).

Although a vast array of drugs is currently available for a variety of pathologies, some of them exhibit bioavailability problems that limit their therapeutic potential. Factors such as reduced solubility, low permeability, reduced lifetime and low stability of some drugs, make their formulation a challenging task (Swaminathan et al., 2016; Babadi et al., 2021). CDNSs, as a result of their spongy structure, have the ability to form inclusion and non-inclusion complexes with different drugs, and thus constitute an effective therapeutic vehicle for the delivery of drugs with low bioavailability. The cavity of the CDs allows the inclusion of hydrophobic molecules and the more hydrophilic outer polymeric network is able to accommodate less lipophilic molecules. Furthermore, CDNSs combine high biocompatibility, biodegradability and low cytotoxicity, conferred by the cyclodextrins, with high thermal stability and insolubility that arises from the fact that they are highly crosslinked polymers (Tejashri et al., 2013; Shende P. et al., 2015; Krabicová et al., 2020; Deng et al., 2021; Mashaqbeh et al., 2021). Thus, it is not surprising that this type of nanomaterials has been explored for the oral, topical and parenteral delivery of numerous drugs, in controlled drug delivery systems as, for example, for target cancer therapy (Allahyari et al., 2022). It is worth noticing that the same ability and selectivity of CD towards drug encapsulation and delivery can be used for drug scavengers. In fact, the use of βCD-NS as reactive oxygen species scanvanger has been reported by Kumar and Rao (2022).

Caldera et al. (Caldera et al., 2017) categorized CDNSs into four generations, according to their chemical composition and properties, which also represent the evolution of these systems in pharmaceutical applications. First generation CDNSs are synthesized by reacting CDs with a crosslinking agent (diisocyanate, active carbonyl compounds, anhydrides, polyacids or epoxides), leading to the formation of NSs containing urethane, carbonate, ester and ether functionalities. Among these, CD-based NSs containing carbonate and ester functionalities are the most used in pharmaceutical applications. This type of nanomaterials has been used in the oral delivery of drugs with low solubility and/or high degradability, such as ferulic (antioxidant, anticancer) (Rezaei et al., 2019) and kynurenic acids (antioxidant, neuroprotector) (Dhakar et al., 2019) paliperidone (antipsychotic) (Sherje et al., 2019), tamoxifen (anticancer) (Torne et al., 2013), paclitaxel (anticancer) (Torne et al., 2010; Ansari et al., 2011a; Mognetti et al., 2012), rosuvastatin (antipsychotic) (Gabr et al., 2018), repaglinide (antidiabetic) (Olteanu et al., 2014), rilpivirine (antiretroviral) (Rao et al., 2018), norfloxacin (antibiotic) (Mendes et al., 2018), erlotinib (anticancer) (Dora et al., 2016), resveratrol (anti-inflammatory, antioxidant, anticancer) (Ansari et al., 2011b; Pushpalatha et al., 2018a) curcumin (anti-inflammatory, antioxidant, anticancer) (Pushpalatha et al., 2018b; Möller et al., 2021) camptothecin (antitumor) (Swaminathan et al., 2010b; Minelli et al., 2012; Gigliotti et al., 2016, 2017) and dithranol (drug for psoriasis) (Kumar S. and Rao R., 2021), among others.

The experimental results obtained show that the solubility/degradability of drugs is improved when they are incorporated into NSs, thus increasing their bioavailability. In the case of drugs used in the cancer treatment, there is generally a reduction in side effects and toxicity of these drugs and greater tumor inhibition, when compared to the free drug (Trotta et al., 2012, 2014).

Recently, Pivato et al. have reported the synthesis of a βCDNS hydrogel using pyromellitic dianhydride as crosslinker. The loading and release of piroxicam showed that a two-step release kinetics is occurring: at short-range times the kinetics is characterized by a pseudo-Fickian mechanism, whilst for long times the release is dominated by the drug previously encapsulated in the CD cavities. This 3D material will offer a sustained release of drug over 75 h (Pivato et al., 2021).

The interaction of CDNSs with drugs and the stability of formulations is usually confirmed using various analytical techniques, including zeta potential, FTIR, DSC, and XRD. The determination of zeta potentials allows to validate the stability of the prepared formulations. The interaction of drugs with NSs can be confirmed using FTIR, by the presence of characteristic drug peaks in loaded NSs and by the shift observed in those peaks when the drug is incorporated into NSs. DSC thermograms often show a reduction/suppression or shift in drug crystalline peaks after encapsulation due to the formation of inclusion complexes. XRD studies show that, in general, crystalline drugs adopt an amorphous form when complexation with the NSs occurs (Shende P. K. et al., 2015; Pushpalatha et al., 2018a; Allahyari et al., 2020, 2021; Pawar and Shende, 2021; Suvarna et al., 2021).

One of the major problems concerning conventional drug delivery is that the concentration of drug in the bloodstream reaches a maximum, followed by a sharp decrease. Thus, the concentration of the drug in the body is not stable, which reduces its effectiveness. The use of controlled release systems makes it possible to maintain more stable drug levels, reducing the frequency of doses and the side effects that result from dose peaks. NSs have shown to be promising materials in this field (Subramanian et al., 2012; Menezes et al., 2019). In particular, CDNSs have been used for the controlled release of some drugs, including meloxicam (anti-inflammatory) (Shende P. K. et al., 2015), nifedipine (antihypertensive) (Shringirishi et al., 2017), telmisartan (antihypertensive) (Rao et al., 2013) and curcumin (Darandale and Vavia, 2013; Gholibegloo et al., 2019b; Rafati et al., 2019).

Other relevant applications in pharmaceuticals include the dermal transport of active principles, such as diclofenac (anti-inflammatory) (Conte et al., 2014), imiquimod (antitumor) (Argenziano et al., 2020a) or curcumin and resveratrol (Pushpalatha et al., 2019), the encapsulation of essential oils which exhibit antioxidant and antimicrobial activity (Nait Bachir et al., 2019; Silva et al., 2019; Simionato et al., 2019), and oxygen delivery (Coviello et al., 2017). Table 3 summarizes recent examples of the use of first generation CDNSs in pharmaceutical applications.

**TABLE 3:** Some recent examples (2020–2021) of first generation CDNSs applications in pharmaceuticals.

Nanosponge	Drug	Application	Main results
α-, βCD:CDI Matencio et al. (2020a)	Oxyresveratrol	Anticancer drug delivery	Strong cell viability inhibition for HT-29 and HCT-116 cancer cell lines
βCD:PMA; βCD:DPC Suvarna et al. (2021)	Irbesartan	Solubility enhancement	PMA crosslinker enhanced the drug solubility (81.86 folds) and drug release to a greater extent than DPC crosslinker; encapsulation efficiency up to 38%
βCD:PMA Appleton et al. (2020)	Insulin	Protein delivery	Loading capability 14%; encapsulation efficiency >90%; *In vitro* release of insulin negligible at a gastric pH (<2%) and sustained at intestinal pH
HPβCD + βCD:CDI Pawar and Shende (2020b)	Artemether Lumefantrine	Drug delivery	Entrapment efficiencies of 70.6% for artemether and 88.3% for lumefantrine; *In-vitro* release study showed controlled-release of actives up to 24 h; *In vitro* antimalarial study showed significant action towards RKL-9 strains in comparison to MRC-2 strains
βCD:DPC Kumar et al. (2021)	Clobetasol propionate	Topical delivery	Drug release 86.25%; Appreciable anti-psoriatic activity and alleviated severity of side effects
βCD:DPC Gupta et al. (2021)	Sesamol	Photostability enhancement	Encapsulation efficiency 90.66%; Enhancement of stability, while retaining antioxidant and anti-tyrosinase potential
βCD:DPC Amin et al. (2020)	Febuxostat	Oral bioavailability enhancement	≥30% release at first hour followed by controlled release (≥75%) at 6 h
βCD:DMC Moin et al. (2020)	Paracetamol + aceclofenac + caffeine	Solubility/combination therapy enhancement	*In vitro* studies indicate rapid dissolution compared to pure drugs; Formulation stable up to 45 days
βCD:CDI Srivastava et al. (2021)	Econazole nitrate	Topical delivery	Entrapment efficiency 70.13%; nanogel was able to impede the fungal growth both *in vitro* and *in vivo*
βCD:CDI Yaşayan et al. (2020)	Sulfamethoxazole	Solubility enhancement	Improved solubility up to 30-fold
βCD:PMA Clemente et al. (2020)	ICOS-Fc	Cancer therapy	*In vivo* experiments showed that treatment of C57BL6/J mice with ICOS-Fc^a^ loaded in CDNPs inhibits the growth of subcutaneous B16-F10 tumors
βCD:CDI Allahyari et al. (2021)	flutamide	Anticancer drug delivery	Increased dissolution rate, sustained release and considerable uptake into PC3 cell line was observed
βCD:CDI Allahyari et al. (2020)	Bortezomib	Anticancer drug delivery	Uptake of 93.9% in 3 h against MCF-7 cell line; Higher IC_50_ in comparison with the plain drug
βCD:PMA Argenziano et al. (2020b)	Doxorubicin	Cancer therapy	Higher accumulation in the tumor and neoplastic cells; Reduced cardiotoxicity
βCD:DPC Rezaei et al. (2021)	Thyme essential oil	Solubility and volatility reduction enhancement	Increased antibacterial activity; Aqueous solubility enhanced 15-folds; Minimum inhibitory concentration decreased up to 29.4 fold after encapsulation
βCD:DPC Salehi et al. (2021)	Limonene essential oil	Solubility and volatility reduction enhancement	Enhanced solubility and thermal stability; Higher antibacterial activity; Minimum inhibitory decreased after encapsulation
βCD:DMC Iriventi et al. (2020)	curcumin + caffeine	Topical delivery	69.72% *in vitro* drug release; Promising anti-psoriatic activity

a ICOS-Fc: soluble recombinant form of inducible T-cell costimulatory.

In second generation of CDNSs other functional groups, in addition to those already existing in the CDs or in the crosslinker, are introduced through functionalization before, after or concomitantly with the crosslinking. This functionalization allows to modulate the polarity or charge of the NSs, according to the characteristics of the drugs to be encapsulated, or the coupling of fluorescent compounds, which can be useful for diagnosis and cancer therapy (Trotta, 2011; Mognetti et al., 2012; Caldera et al., 2017). For example, the use of negatively charged NSs, obtained by reacting βCD with succinic anhydride and functionalized with fluorescein isothiocyanate, were used for acyclovir delivery. *In vitro* studies showed prolonged drug release kinetics and increased antiviral activity. The coupling of fluorescein allowed to observe the internalization of CDNSs in cells (Lembo et al., 2013). CDNSs prepared by condensation of βCD with 1‒(3‒dimethylaminopropyl)‒3‒ethylcarbodiimide hydrochloride (AMD) and modified with fluorescent compounds (carbon quantum dots) (Pei et al., 2018) and CDNSs (obtained using βCD and Epiclon B-4400 as crosslinker) anchored on magnetite nanoparticles and modified with folic acid (Gholibegloo et al., 2019a) have also been described and used in tumor theranostics, with good results. Another paper refers the use of CDNSs modified with carboxylate groups and impregnated with lysozyme with the aim of being used as antibacterial agents (Deshmukh et al., 2016).

Recent examples of the application of second generation CDNSs in pharmaceuticals include the use of CDNSs, functionalized with cholesterol hydrogen succinate (CHS), as site-specific drug delivery carriers. Doxorubicin was used as model drug and it was observed that modification with CHS increased drug absorption and improved the uptake into the cells (Singh et al., 2018). Another recent study showed that the use of CDNSs functionalized with gold NPs constitutes an efficient drug transport system, with high loading capacities for phenylethylamine and 2-amino-4-(4-chlorophenyl)-thiazole (90 and 150%, respectively), eight times higher than those obtained with native βCD (Asela et al., 2021).

The third generation CDNSs can be categorized as responsive nanomaterials, whose behavior depends on external stimuli such as variations in pH, temperature, light, redox potential or electromagnetic field. (Caldera et al., 2017). NSs of this type can be very useful for controlled drug release induced by a specific stimulus (Krabicová et al., 2020). For example, glutathione-responsive nanosponges were developed by Trota *et al.* (Trotta et al., 2016b; Daga et al., 2016) and prepared by reaction of βCD with 2-HEDS and PMA. The NSs were loaded with a model anticancer drug, doxorubicin, and drug release was observed to be dependent on the glutathione content present in the tumor cells. This same system was also used for the delivery of erlotinib hydrochloride and resveratrol, two drugs used in cancer therapy (Momin et al., 2018; Palminteri et al., 2021), and their *in vitro* biological effects in HCT116, HT‒29, DU145, and PC-3 cancer cell lines was also assessed (Argenziano et al., 2020a). Following a similar approach a dual glutathione/pH dual responsive CDNS was reported (Dai et al., 2021). Another type of pH-responsive NS was obtained by co-polymerization between CD and calixarene derivatives, covalently linked by triazole units (CyCaNSs). CyCaNSs are useful in environmental remediation (Cataldo et al., 2021) and in drug delivery, as the triazole acts actively as antibacterial, antifungal, antitumor and cytotoxic agent (Lo Meo et al., 2014; Pawar et al., 2019).

CDNSs prepared by molecular imprinting techniques allow the preparation of materials that exhibit high selectivity for specific molecules, being considered the fourth generation of NSs (Caldera et al., 2017; Ciesielska et al., 2020; Jain et al., 2020; Krabicová et al., 2020). Work by Trotta *et al.* reports the preparation of MIP-CDNSs by reacting βCD with CDI, in the presence of the template, l-Dopa, a prodrug used in the treatment of Parkinson’s disease. The release of l-Dopa using the synthesized MIP-CDNSs was studied and the results obtained showed good encapsulation efficiencies and a slow and prolonged release of the drug, as a result of the strong interactions established between the drug, the polymeric network of NS and the cavity of the CD (Trotta et al., 2016a).

Beside pharmaceutical applications, cyclodextrin‒based nanosponges have found a growing interest for application in the biomedical field. In this context, βCD‒based materials prepared using PMA as crosslinker seems to be the most applied. For example, βCD:PMA at molar ratio 1:12 found application in health promoting and anti‒aging studies by release of oxyresveratrol (OXY), a compound with antioxidant activity. The study showed an increase of 9.6% in life expectancy *in vitro* using *Caenorhabditis elegans* as animal model (Matencio et al., 2020b). A different biomedical application of βCD:PMA microparticles is as probe for glucose estimation via molecular and non‒molecular imprinting. The molecular imprinting βCD:PMA, synthesized by reacting βCD with PMA and using glucose as the template, present a porous structure and a surface area ranging from 70 to 52 m^2^ g^−1^ and shows an extremely high glucose binding capacity (95%) (Deshmukh et al., 2015).

The volume of studies already carried out using CDNSs shows that these systems are very promising for pharmaceutical and biomedical applications, especially for the delivery and controlled release of drugs. *In vitro* and *in vivo* studies show higher bioavailability of drugs encapsulated in CDNSs when compared to non-encapsulated ones. However, further studies are needed regarding the stability and toxicity of CDNSs and their *in vivo* degradation products (Lembo et al., 2013; Menezes et al., 2019).

### Environmental Remediation

The 3D-structure of CDNSs shows several advantages in their application in environmental remediation, with removal percentages of pollutants similar to those found for activated carbon (AC). Despite the high cost of AC production, CDs and their derivatives are getting cheaper and of easy functionalization, allowing the synthesis of a wide range of highly structured sorbent materials. Moreover, taking advantage of the properties of CDNSs, three different sorption mechanisms can occur simultaneously; they are: 1) the formation of host‒guest complexes by taking advantage of the hydrophobic cavities of CDs; 2) diffusion through pores and channels of the hydrophilic network; and 3) interaction at active sites on the NS surface. Hence, their application in environmental remediation processes lead to remarkable results (Kumari et al., 2020). Moreover, studies have demonstrated that different synthetic conditions, including solvents, aliphatic or aromatic linkers and CDs:crosslinker molar ratio, affect the physico‒chemical properties of the material and thus the removal efficiency of pollutants.

Moreover, considering the monomers used for CD coupling in environmental remediation, EPI is one of the most used aliphatic linkers along with citric acid, HDI and EDTA. As common examples of aromatic linkers we can mention TDI and tetrafluoroterephtalonitrile (TFP).

In this section, we evaluate and compare the role of NSs’ physico‒chemical properties on the sorption mechanisms and removal efficiencies for five different classes of pollutants: heavy metals, pesticides, pharmaceuticals and other aromatic compounds (biphenyls).

#### Heavy Metals

Heavy metals constitute one of the major classes of pollutant worldwide because of their easy absorption by living organisms leading to bioaugmentation and bioaccumulation. Particularly, the exposure to heavy metals can produce allergic reactions, mental disability, dementia, vision problem as well as liver and kidney diseases (Vareda et al., 2016) (Vareda et al., 2019).

CDNSs prepared by using either aliphatic or aromatic crosslinkers for the removal of a broad range of heavy metals (e.g., Cu(II), Zn(II), Pb(II), Cd(II), Ni(II), Co(II), Hg(II), Fe(III), and Cr(III), and As(V)) have been synthesised and tested. Table 4 summarizes the most relevant data on the NSs and sorption parameters.

**TABLE 4:** Recap of the main results of heavy metals removal by different nanosponge formulations.

Nanosponge	Pore size/(nm)	Surface area/(m^2^ g^−1^)	Heavy metal*/q*_*m*_ (mg g^−1^)/RE%	q_m_/(mg g^−1^)	Removal efficiency (RE%)	References
βCD:CA (1:8)^a^	n.a	n.a	n.a./20–70 (high conc.); 80 (Cu(II), Zn(II) at low conc.); 80, 60 (Cu(II), Zn(II) in artificial sea water)	n.a	20–70 (high conc.); 80 (Cu(II), Zn(II) at low conc.); 80, 60 (Cu(II), Zn(II) in artificial sea water)	Rubin Pedrazzo et al. (2019)
βCD:EPI (1:6)	n.a	n.a	Cu(II)/23/≥ 90 in water; Cd(II)/43/≥ 90 in water			Zhao et al. (2015)
βCD:EDTA (1:17)	n.a	n.a	Cu(II)/79/95 in a model textile effluent			
			Cd(II)/124/95 in a model textile effluent			
βCD:PMA (1:8)	n.a	n.a	n.a./20–70 (high conc.); 80 (Cu(II), Zn(II) at low conc.); 80, 60 (Cu(II), Zn(II) in artificial sea water)	n.a	20–70 (high conc.); 80 (Cu(II), Zn(II) at low conc.); 80, 60 (Cu(II), Zn(II) in artificial sea water)	Rubin Pedrazzo et al. (2019)
β-MCD:VI (1:100)	0.02	27.5	Pb(II)/18/n.a			Qin et al. (2019)
			Cu(II)/55/n.a			
			Cd(II)/65/n.a			
			Zn(II)/50/n.a			
			Ni(II)/25/n.a			
			Co(II)/20/n.a			
βCD:TFP (1:3)	3	271	Pb(II)/196/70			He et al. (2017)
			Cu(II)/164/77			
			Cd(II)/136/83			
βCD:TDI (1:10)	1.6	2.4	As(V)/n.a./≈10			Raoov et al. (2014)
βBZMCD:TDI (1:10)	77.6	1.3	As(V)/n.a./≈95			
βCD:TPC:TA (1:4)	<10	2.3	Pb(II)/136/≈90			Yang et al. (2022)

a Molar ratio values in mol/mol.

Concerning aliphatic-based CDNSs, citric acid (Rubin Pedrazzo et al., 2019) and EDTA (Zhao et al., 2015) are relevant crosslinkers, not only because they form highly crosslinked materials but also due to the number of available carboxylic acid groups, which allows a significant increase in active sites for interaction with metal ions. Moreover, the sorption ability of these materials is pH responsive. βCD:EDTA shows the highest removal efficiency for Cd(II) and Cu(II) reaching values higher than 90% (Zhao et al., 2015).

A different approach is the modification of chitosan by EDTA, followed by the crosslinking to βCD using pentafluoropyridine (βCD‒PFP‒CTS/EDTA). This βCD‒PFP‒CTS/EDTA NS exploits the simultaneous chelating ability of CTS and EDTA. As for pure βCD‒EDTA, the sorption process is promoted at acidic pH with a removal higher than 90% for Pb(II), Ni(II), Cu(II), Co(II), Hg(II) and Cr(III) in about 1 min (Yu et al., 2018b). Likewise, the removal of Pb(II), Cu(II) and Cd(II) was studied by using NSs of βCD and TFP and methacrylic-βCD with 1‒vinylimidazole (VI) (MCD:VI). βCD:TFP exhibits the largest active surface area which plays a key role on the significant amount of heavy metals sorbed per gram of material (Pb(II): 196.4 mg g^−1^, Cu(II): 164.4 mg g^−1^ and Cd(II): 136.4 mg g^−1^). However, its removal efficiency diminishes along cycles of sorption/desorption (He et al., 2017). The pH responsive MCD:VI adsorbent was synthesized at different molar ratios. At 1:100 mol/mol, it has revealed the best sorbent performance for Pb(II), Cu(II), Cd(II), Zn(II), Ni(II) and Co(II). The sorption ability of MCD:VI via coordinative interaction decreases in the order Cd(II)>Cu(II)>Zn(II)>Ni(II)>Co(II)>Pb(II) within 70–15 mg g^−1^ (Qin et al., 2019). Moreover, βCD crosslinked with pyromellitic dianhydride (βCD:PMA) was tested for Cu(II), Zn(II), Pb(II), Cd(II) and Fe(III) removal and the results were compared to those obtained for βCD:CA. Both materials show similar removal efficiencies (from 20 to 70%), however, the highest removal efficiencies (ca. 80%) are obtained for initial low concentrations of Cu(II) and Zn(II) (Rubin Pedrazzo et al., 2019).

Toluene diisocyanate was used to synthesize βCD and (6‒deoxy)‒(6‒benzylimidazolium)‒βCD NSs. The latter shows higher efficiency in the removal of metal ions, due to electrostatic interactions and chelating ability of the imidazolium groups, as well as higher thermal stability (Raoov et al., 2014). The hydroxyl-rich tannic acid (TA) is an interesting monomer used to synthesise βCD-TA nanosponge crosslinked by terephthaloyl chloride (TPC) and applied for Pb(II) removal. βCD:TPC:TA shows a selective and high removal efficiency of 97% for Pb(II) at alkaline pH, in the presence of salt, humic acid and other interferents (Yang et al., 2022).

#### Dyes

Dyes are used in many industries, mainly involving the production of consumables, including textiles, inks and paper. The textile industry is one of the activities that consumes greatest amounts of water, either in quality or in quantity, associated with the use of dyes, which makes this industry one of the biggest water body pollutants. Moreover, dyes are, in general, water soluble compounds, making them difficult to remove from wastewaters (Lellis et al., 2019). Being a relevant issue, several CDNSs have shown effectiveness in removing dyes (Table 5). For instance, βCD:EPI macroparticles show a spontaneous and exothermic sorption of DirectBlue78 (Murcia-Salvador et al., 2019). The effect of other cyclodextrins, αCD and hydroxypropyl-αCD, on the synthesis of NSs and consequent performance on Direct Red83:1 dye removal has been evaluated. The removal efficiency (*RE*) and the maximum amount sorbed (*q*_*m*_) of *RE* = 92.8% and *q*_*m*_ = 31.5 mg g^−1^ and *RE* = 75% and *q*_*m*_ = 23.41 mg g^−1^, for αCD:EPI and HP-αCD:EPI, respectively, have been obtained (Pellicer et al., 2018).

**TABLE 5:** Selected examples of nanosponges application for dye removal.

Nanosponge^a^	Pore size/(nm)	Surface area/(m^2^ g^−1^)	Dye/qm (mg g^−1^)/RE (%)	References
βCD:EPI (1:135)^b^	n.a	n.a	DirectBlue 78/24/n.a	Murcia-Salvador et al. (2019)
αCD:EPI (1:115)	n.a	n.a	DirectRed83:1/32/93	Pellicer et al. (2018)
HP-αCD:EPI	n.a	n.a	DirectRed83:1/23/75	
βCD:EPI (1:6)	n.a	n.a	Methylene blue/50/≥ 90; Safranin/6/≥ 90; Crystal violet/42/≥ 90	Zhao et al. (2015)
βCD:EDTA (1:17)	n.a	n.a	Methylene blue/84/n.a.; Safranin/60/n.a.; Crystal violet/114/n.a	
MCD:MPP (1:1)	2–10	n.a	Rhodamine B/10/n.a.; Congo Red	Qin et al. (2019)
β-MCD:VI (1:100)	0.02	28	Rhodamine B/175/60; Congo red/712/100	Qin et al. (2019)
β-BCD:PD	no porous	22	Methyl orange/285/77; Congo red/288/80; Rhodamine B/n.a./n.a.; Methylene blue/n.a./n.a	Li et al. (2018b)
βCD‒P5 (1:1)	4	479	Methylene blue/135/78	Lu et al. (2019)
βCD:FPS (1:2)	5–6	n.a	2‒naphthol/n.a./>80	Wang et al. (2017)
			2‒naphthol/n.a./>99 (flow through)	
βCD:CMP(H)	1–10	1,099	nitrobenzene/325/80	Huang et al. (2020)
			2‒nitrophenol/310/75	
			2‒nitroaniline/290/70	
			4‒nitroaniline/275/65	
			2‒chloroaniline/300/75	

a MPP [2,2′‒azobis (2‒methylpropionitrile)].

b Values of molar ratio in mol/mol.

The βCD:EDTA was also applied for the removal of different dyes (methylene blue (MB), safranin O (SF) and crystal violet (CV)), highlighting the removal efficiency higher than 90% for the MB. As mentioned above, the presence of carboxylate groups in EDTA are relevant for the interaction with dye molecules (Zhao et al., 2015).

On the other hand, MCD:VI obtained via free radical copolymerization at different molar ratios (from 1:10 to 1:150 mol/mol) was tested for sorption of rhodamine B (RB) and Congo red (CR); it has been demonstrated that pH strongly influences the electrostatic and *π*‒*π* interactions occurring between NSs and dye molecules. Moreover the affinity of dyes towards MCD depends on the VI molar ratio; i.e., for CR the *q*_*m*_ increases by increasing the molar ratio, reaching a maximum *q*_*m*_ value (1.12 mg g^−1^) for MCD:VI150 and for RB, the opposite is observed (the highest *q*_*m*_ value is obtained for MCD:VI10, 336 mg g^−1^) (Qin et al., 2019).

The effect of benzyl-βCD hyperlinked with 4,4′-bipyridine (PD), synthesised through the Menshutkin reaction, in the sorption of two cationic (RB and MB) and two anionic (CR and methyl orange, MO) dyes has been reported by Li et al. This NS is effective in the removal of anionic dyes: RE = 80 and 77% for CR and MO, respectively (Li X. et al., 2018).

MB is a molecule used very often as a drug and dye model. Therefore, the interaction between MB and NS goes beyond the interest in the sorption of MB onto NSs. The copolymerization of two molecules containing cavity gates [the βCD and the pillar (5)arene (P5)], using TFP as crosslinker, provides the ability to form two different types of host‒guest complexes as well as to manage the hydrophilic/hydrophobic balance of the NS. Lu et al. (Lu et al., 2019) have found that by increasing the molar fraction of P5 the hydrophobicity increases and the best removal efficiency of MB is attained (78%).

Different classes of dye intermediates and dyes derived from benzene were studied using two uncommon linkers: the 4,4′‒difluorodiphenylsulfone (FPS) and the 4,4′-bis(chloromethyl)-1,1′-biphenyl (CMP) for βCD and βBCD, respectively. βCD:FPS was tested for 2-naphthol removal allowing >80 and 99% removal efficiency for in batch and in flow-through sorption experiments, respectively (Wang et al., 2017). On the other hand, the adsorption of different aromatic compounds, such as nitrobenzene, 2-nitrophenol, 2-nitroaniline, 4-nitroaniline and 2‒chloroaniline, onto βCD:CMP has also been evaluated. It is worth mentioning that, for this NS, the percentage of crosslinker significantly modifies the active surface area; i.e., an increase in the crosslinking degree may result in an increase in the surface area up to 5 times. Thus, surface area and the phenyls groups play a crucial role on the sorption efficiency by enhancing the π‒π interactions with removal efficiency >70% for any dye intermediates (Huang et al., 2020).

#### Pesticides

Nowadays pesticides are one of the most concerning classes of substances related with environmental pollution and human health issues. Recently, nanosponges have been considered promising sorbent materials and they were applied for removal of pesticides and their intermediates such as, atrazine, benalaxyl, bromacil, butachlor, butylene fipronil, fenamiphos, fipronil, fluprifole, fomesafen, pretilachlor, simazine, 4‒*n*‒nonylphenol (4*n*NP), 4‒*n*‒octylphenol (4*n*OP) and 4‒*tert*‒octyphenol (4*t*OP) (Table 6).

**TABLE 6:** The main types of CDNSs applied for the adsorption of pesticides.

Nanosponge	Pore size/(nm)	Surface area/(m^2^ g^−1^)	Pesticide/*q*_*m*_ (mg g^−1^)/RE %	References
αCD:EPI (1:115)^a^	n.a	n.a	Atrazine/≈0.07/≈60	Romita et al. (2019)
βCD:EPI (1:115)	n.a	n.a	Atrazine/≈0.07/≈60	Romita et al. (2019)
Multiplex	5	1–4	Atrazine/10/30	Liu et al. (2011)
			Benalaxyl/23/65	
			Bromacil/11/25	
			Butachlor 115/85; butene fipronil/6/98	
			fenamiphos/21/60	
			fipronil/8/85	
			fomesafen/25/25	
			pretilachlor/83/55	
			simazine/1/28	
βCD:EPI/KMnO_4_	4	1	Atrazine/n.a./17	Wang et al. (2021b)
			Benalaxyl/n.a./70	
			Bromacil/n.a./19	
			Butachlor/n.a./80	
			Fenamiphos/n.a./58	
			Fipronil/n.a./48	
			Flufiprole/n.a./80	
			Pretilachlor/n.a./40	
βCD:am_6_ (1:20)	24	22	Imidacloprid/68/97	Utzeri et al. (2022)
BZM-βCD:TDI (1:10)	78	1	2‒chlorophenol/n.a./25	Raoov et al. (2014)
			4‒chloro‒3‒methylphenol/n.a./90	
			4‒nitrophenol/n.a./80	
			2,4,6‒trichlorophenol/n.a./85	
			2‒nitrophenol/n.a./70	
			2,4‒dinitrophenol/n.a./85	
			2,4‒dichlorophenol/n.a./70	
βCD:TTI3% (1:2)	n.a	34	2,4‒dichlorophenol/145/68	Zhou et al. (2019a)
βCD:TFP (1:3)	2–4	263	2,4‒dichlorophenol/14/85	Alsbaiee et al. (2016)
			1‒naphtyl amine/13/92	
			Metolachlor/26/92	
βCD:TFP:THTS (1:1:2)	2–25	231	2,4‒dichlorophenol/≈500/≈90	Wang et al. (2019b)
βCD:DPC (1:4)	2	3	4‒chlorophenoxyacetic acid/1/91	Salazar et al. (2018)
			2,3,4,6‒tetrachlorophenol/1/78	
αCD*/*βCD/γCD:DCO	non-porous	n.a	Carbendazim/0.3/90 (after 5 h)	Rizzi et al. (2021)

a Molar ratio values in mol/mol.

Macroparticles of αCD:EPI, βCD:EPI and γCD:EPI, with diameter ranging from 0.212 to 0.250 mm, were used for atrazine sorption. αCD:EPI and βCD:EPI have similar removal efficiencies (around 60%) with sorption isotherms characterized by the Freundlich model, whilst the RE of γCD:EPI is only 50%, in agreement with the highest ratio between the volume of γCD cavity and the aromatic ring of atrazine (Romita et al., 2019). Additionally, atrazine, benalaxyl, bromacil, butachlor, butylene fipronil, fenamiphos, fipronil, fomesafen, pretilachlor and simazine were tested in a screening study of sorption ability of βCD:EPI, *γ*CD:EPI, HPβCD:EPI, randomly methylated βCD:EPI (RM-βCD:EPI), equimolar mixture of β-γ-CD:EPI, βHPβCD:EPI and γHPβCD:EPI and a physical mixture of βCD:EPI, HPβCD:EPI and RM-βCD:EPI with mass ratio of 40:30:30 (w/w) (multiplex). Multiplex NSs combining the physico‒chemical properties of the three materials, show the best performance as sorbent material, even at low concentrations either in deionized water or in sea water, even after five cycles of regeneration (Liu et al., 2011). In a similar way βCD:EPI and γCD:EPI were initially synthesised and, subsequently, oxidized with KMnO_4_. Albeit swelling ratio, particle and pore sizes, the crosslinking degree and hydrophilicity increase by increasing the amount of KMnO_4_, while the CD content and surface area decrease concomitantly. It has also be found that γCD:EPI is more efficient for hydrophobic compound removal (*i.e.* benalaxyl, butachlor, fipronil and flufiprole) whereas βCD:EPI performs better for hydrophilic pesticides (i.e. atrazine, bromacil, fenamiphos, pretilachlor) (Wang M. et al., 2021). The sorption experiments involving 4*n*NP, 4*n*OP and 4*t*OP and CD-based:EPI NSs, where the mixture of α- β- and γCD NS, using EPI as crosslinker, stands out, have shown high removal efficiencies for all the adsorbates. In general, all sorbents have *RE* around 90%. However, the HP-βCD shows the highest performance, in the order 4*t*OP>4*n*NP≈4*n*OP, due to its higher hydrophilicity and host‒guest complex formation (Crini et al., 2021).

In a recent study, βCDNSs were synthesized using as linker two diamine monomers: 1,6‒hexane diamine and 1,12‒dodecane diamine. Physico‒chemical properties were characterized and their influence on the sorption process assessed. Both materials present similar particle size (430 nm) but the CD-am_6_ shows lower thermal stability but higher hydrophilicity, surface area and pore size. These properties seem to have relevance for the highest removal efficiency of imidacloprid (>90%), obtained for initial concentrations of IMD ranging from 20 to 300 mg L^−1^ (Utzeri et al., 2022). On the other hand, for βCD crosslinked with hexamethylene diisocyanate (βCD:HDI) the removal efficiencies for imidacloprid and cymoxanil were low; however, by forming composite gels with just 10% (w/w) of AC the removal efficiency increases to 81 and 73%, respectively (Utzeri et al., 2021).

Spherical βCD:HDI (10 equivalents of crosslinker) with high thermal stability was applied for the removal of 2,4‒dinitrophenol (2,4NP) in different environmental water samples (tap water, lake water, river water and sea water). A higher removal efficiency (ca. 74%), at acidic pH and low analyte concentration, was observed; the RE remained constant along five sorption/desorption cycles (Anne et al., 2018). In the same study, the performance of βCD:TDI for the removal of 2,4NP has been compared with that obtained by using βCD:HDI. The porous βCD:TDI presents higher efficiency (ca. 85%) than the non-porous βCD:HDI. However, results can also be justified by considering the presence of the phenyl groups of the linkers, which can form *π*‒*π* interactions with 2,4-dichlorophenoxy acid (2,4D) (Anne et al., 2018).

As was pointed out in the previous section, β-BZMCD:TDI was applied for the removal of a set of phenols with different substitutions. The β-BZMCD:TDI shows higher ability than the non-substituted βCD:TDI, with an average removal percentage equal to 85%, which can be justified by its higher porosity (Raoov et al., 2014). The sorption of 2,4CP onto βCD:TTI (triphenylmethane‒4,4′,4″‒trisocyanate), synthesised using 2‒butanone as solvent, has been studied. The properties of the NS is highly affected by the catalyst/linker ratio, temperature and βCD:TTI molar ratio (Zhou L. et al., 2019). Another example of a common aromatic linker is TFP. A study comparing the sorbent performance of porous and non-porous βCD:TFP has also been reported. Sorption tests were performed with 2,4CP, 1-naphtyl amine and metolachlor. Porous βCD:TFP has a lower water uptake, compared with non-porous material, but has shown better removal efficiency ≥85% (Alsbaiee et al., 2016). Pure βCD:TFP was also used as control material in comparison with a mixture of two monomers, TFP and 5,5′,6,6′‒tetrahydroxy-3,3,3′,3′-tetramethylspirobisindande (THTS), prepared in dimethylacetamide. Overall, NS particles exhibit dimensions ca. 90 nm diameter with micro‒ and meso‒pores and higher active surface area; these properties are improved by increasing the THTS percentage. The βCD:TFP:THTS0.5 is the most efficient material for various micropollutants. Particularly, it sorbs 460 mg g^−1^ of 2,4CP per gram of sorbent corresponding to around 90% of removal efficiency. βCD:P5 at molar ratio 1:1 presents the best hydrophilic behaviour, higher surface area (479 m^2^ g^−1^) than βCD:TFP and larger pore size (3.5 nm) than P5:TFP. They represent an advantage on sorption processes of 1‒naphthylamine with removal efficiency of around 80% in aqueous solution and environmental conditions (Lu et al., 2019). An interesting study reported by Klemes et al. shows how solvent, catalyst (K_2_CO_3_) and crosslinker ratio influence both reaction yield and physico‒chemical properties of βCD:TFP NS. Higher TFP amount led to a decrease in reaction yield and phenolate incorporation. Moreover, depending on the method of addition of K_2_CO_3_, a porous material may result, with 346 m^2^ g^−1^ of active surface, or even a non‒porous material. NSs obtained using different methods were tested towards 83 pesticides. In general, a stronger affinity of the micropollutants was observed for the porous βCD:TFP synthesised by using DMSO, with higher surface area and phenolate content (Klemes et al., 2018). Another extensive study involving βCD:TFP, was carried out by Li *et al.* (Li C. et al., 2018). It has been demonstrated that for almost all pesticides the removal efficiency of the NS is higher than 70%.

NSs based on aromatic linkers such as FPS, CMP and DPC have also been prepared and their performance towards pesticide sorption evaluated. The sorption of 2,4CP by βCD:FPS (1:2 mol/mol) showed a removal efficiency higher than 80% (Wang et al., 2017). The aromatic crosslinker can act synergetically in the sorption process of organic adsorbates; for example, the sorption of 2NP, 2,4CP, 2,6CP and 2,4,6CP onto βCD:CMP increases by increasing the amount of crosslinker (Huang et al., 2020).

The incorporation of Fe_3_O_4_ nanoparticles into βCD:DPC for the sorption of aromatic chlorinated pesticides [4‒chlorophenoxyacetic acid (CPA) and 2,3,4,6‒tetrachlorophenol (TCP)] has demonstrated that no variation of removal efficiency occurs; however, the sorption kinetics seem to be facilitated by the presence of NPs (Salazar et al., 2018). The same research group has also tested this composite for dinotefuran removal with similar results (Salazar et al., 2020).

Non‒porous materials with high thermal stability were synthesized using BDE as linker for α-, β- and γCDs. The materials were mainly applied for ciprofloxacin removal, although they were also tested for carbendazim (a broad-spectrum fungicide) with 70% of active substance sorbed in 30 min and a value of 90% can be reached after 5 h sorption (Rizzi et al., 2021).

Hybrid NSs, which include CD and a polymer, have also been synthesised and tested for the removal of pesticides. Recently, poly (vinyl alcohol)-βCD has been synthesised and its adsorption performance evaluated towards paraquat. The obtained material shows a very fast adsorption of pesticide, with a RE of around 96% in just 3 min, following a Langmuir mechanism (Martwong et al., 2021, 2022).

#### Drug Removal

As the other classes of pollutants, pharmaceuticals represent one of the biggest concern in relation with ecosystem pollution and its side effects. In Europe, they were detected in surface and groundwater that are used in agriculture and drinking water production. Thus, strategical actions are made to face this issue. One of these strategies is the development of efficient sorbent materials, including nanosponges (Table 7). βCD:EPI particles were employed in a pilot scale test for the simultaneous removal of eight different drugs (β‒estradiol, ethynyl estradiol, estriol, ibuprofen, diclofenac, naproxen, ketoprofen and cholesterol) from greenhouse municipal wastewater. Particles reach up ca. 99% of removal efficiency for hormones and 85% for ibuprofen and diclofenac, in‒flow sorption process (Fenyvesi et al., 2020). Commercial βCD:EPI macroparticles were used for the removal of twenty one different drugs existing in wastewater effluents. The adsorption process is carried out together with a photodegradation treatment. The results show a removal efficiency of 71% after 5 min for most of the drugs, whereas a 99% removal is reached for metoprolol, paroxetine and propranolol. Generally, the removal efficiency follows the order: β‒blockers > psychiatric drugs > lipid regulators > antibiotics > anti‒inflammatories > diuretics (Gómez-Morte et al., 2021). Additionally, fibers of HP-βCD:EPI have been evaluated as adsorbent for removal of phenanthrene—a compound used for the synthesis of bile acids and steroids. It has been found that these fibers were able to remove 80% of phenantrene (Celebioglu et al., 2019).

**TABLE 7:** Nanosponges developed for drug removal (2015–2019).

Nanosponge	Pore size/(nm)	Surface area/(m^2^ g^−1^)	Drugs/*q*_*m*_ (mg g^−1^)/RE %	References
αCD:CA	n.a	—	Progesterone/n.a./95	Moulahcene et al. (2015)
βCD:CHI (1:1)^a^	n.a	n.a	Testosterone/n.a./98–100; epitestosterone/n.a./98–100; androsterone/n.a./98–100; etiocholanolone/n.a./98–100; 5α‒androstane‒3α,17β‒diol/n.a./98–100; 5β‒androstane‒3α,17β‒diol/n.a./98–100	Manaf et al. (2018)
βCD:PFP:CTS/EDTA (1:2,900)	1	48	6‒bromo‒2‒naphtol/0.30/> 90	Yu et al. (2018b)
βCD:TFP:THTS (1:1:2)	2–25	231	Propranolol hydrochloride/≈100/n.a	Wang et al. (2019b)
βCD:TFP (1:3)	2–4	21	ethynyl oestradiol/n.a./≈60; propranolol hydrochloride/n.a./≈90	Alsbaiee et al. (2016)
	n.a		Chloroxylenol/144/91; Carbamazepine/136/65	Zhou et al. (2019b)
βBCD:CMP	2–5	1,107	Albendazole/180/96	Wang et al. (2019a)
βCD:HDI (1:4)	n.a	n.a	Ibuprofen/86/<70	Skwierawska et al. (2021)

a Values of molar ratio in mol/mol.

Nanosponges of αCD, βCD and γCD crosslinked with citric acid have been evaluated by Moulahcene et al. for the sorption of progesterone. The αCD:CA NS showed the best sorption performance in continuous flow column (95%); that was justified by the lower swelling ability, higher surface area and number of acidic groups (among all three NSs). (Moulahcene et al., 2015).

βCD:EDTA was applied for the removal of ciprofloxacin, a quimolone antibiotic, having reached a maximum sorbed amount of 327 mg g^−1^, at a pH range 4–6. The sorption mechanism is sensitive to the ionic strength, probably due to screening effect caused by the counterions (Yu F. et al., 2018). The ciprofloxacin removal was further studied with αCD, βCD and γCD crosslinked with BDE and the removal efficiency was also quite promising (i.e., 90%). In a similar way, the sorption capacity is impaired by the presence of salts and pH changes (Rizzi et al., 2021).

In an interesting study, βCD nanosponges obtained using aliphatic and aromatic linkers of the diisocyanate class, dicyclohexylmethane-4,4′-diisocyanate (CHI) and MDI, respectively, were compared. Three molar ratios, 1:1, 1:2 and 1:3 mol/mol were used. The polymers present different hydrophilic/hydrophobic ratios. Tests were performed on artificial and human urines to analyse sorption abilities for testosterone, epitestosterone, androsterone, etiocholanolone, 5*α*‒androstane-3*α*,17*β*-diol and 5*β*-androstane-3*α*,17*β*‒diol. βCD:CHI at molar ratio 1:1 showed the best performance for each analyte with accuracy and recovery within 96 and 106%, respectively. The superior performance of βCD:CHI can be justified considering the higher mobility and flexibility of the aliphatic linker, making the active sites more easily accessible (Manaf et al., 2018).

Materials already mentioned in previous sections were also applied for pharmaceutical removal. βCD:TFP has been used for a number of studies involving the adsorption of many drugs. The properties of βCD:TFP are dependent on the solvent and CD:crosslinker molar ratio (Klemes et al., 2018) (Li C. et al., 2018). Among the adsorbates evaluated we can mention ethynyl oestradiol and propranolol hydrochloride (Alsbaiee et al., 2016; Wang Z. et al., 2019), and chloroxylenol and carbamazepine (Zhou Y. et al., 2019). With few exceptions, this NS reached efficiencies higher than 70%, which shows a high affinity of NSs for different kinds of pharmaceutical molecules.

Moreover, four types of nanosponges with different crosslinkers, namely, PMA, CDI, CA and TDI, were synthesised. βCD:PMA and βCD:CA show the higher level of swelling justified by the larger number of donator and acceptor hydrogen bonding. The removal efficiency of the NSs was tested for indole in three different media: water, simulated gastric fluid and intestinal fluid. Indole is produced by tryptophan conversion, and it is a precursor of uremic toxins responsible of several severe health illnesses. The removal ability of the materials for indole decrease in the following order: βCD:TDI (≈91%)>βCD:CA>βCD:PMA>βCD:CDI (Varan et al., 2020).

#### Other Aromatic Compounds

Biphenols are derivatives of phenol used in the manufacture of plastic furniture and are key components of liquid crystal polymers. Nonetheless, due to their high volume of production and usage, they are classified as persistent organic pollutants. They are hazardous for the environment and for human health. Bisphenol A (BPA) is the most produced and thus the most common in water bodies. Consequently, there is a demand for adsorbents capable of removing these pollutants. The aromatic ring of these compounds can strongly interact, via host-guest interaction, with CD, making CDNSs ideal for that purpose. In fact, several CDNS adsorbents were studied and high removal efficiencies were reported. For example, βCD:EPI, βCD:PFP:CTS/EDTA, βCD:P5 and βCD:TFP:THTS have RE greater than 90% in the removal of Bisphenol A (Alsbaiee et al., 2016; Yu et al., 2018c; Wang Z. et al., 2019; Lu et al., 2019).

Other biphenols with less environmental impact were also tested. Thus, the bisphenol S (BPS) (an endocrine disruptor) has a significant interaction with βCD:FPS (1:2 mol/mol) leading to a removal efficiency ≥80% (Wang et al., 2017). The removal of bisphenol F (BPF) and bisphenol AF (BPAF) was also studied by using βCD:CMP. This NS shows a high sorption efficiency due to its larger surface area and a significant number of π‒π interactions (Huang et al., 2020).

Particularly interesting is a study carried out for assessing the adsorption capacity of *heptakis*(2,6‒di‒*O*‒methyl)βCD crosslinked with three different diisocyanates [MDI, 1,4‒phenylene diisocyanate (PDS) and HDI]. Sixty six different biphenyls, from mono to decachlorobiphenyls, using isooctane as solvent, were tested. The highest crosslinked βCD:MDI and βCD:PDS showed adsorption performances around 100%. This behaviour is also dependent on the CD concentration (Kawano et al., 2015).

### Other Applications

#### Pesticide and Fertilizer Delivery System

In a previous section, the ability of CDNSs for the removal of pesticides and other pollutants has been summarized. In this section, we discuss the role of CDs based on a different approach. In order to reduce the amount of pesticides applied in agriculture, the exposure of workers, and to ameliorate the efficiency of the phytopharmaceuticals, a broad range of materials have been developed. Nanosponges of αCD, βCD and γCD were prepared using 1,1′-carbonyldiimidazole (CDI) and PMA as crosslinkers. Both types of nanosponges were loaded in methanol with ailanthone, a natural herbicide characterized by low persistence and rapid degradation. γCD-based materials showed higher interaction for ailanthone with 55% of encapsulation efficiency (EE) and remarkable efficacy on index growth of garden cress and radish after 10 days. The highest EE is found for γCD-based NS, probably due to proper fit between cavity and analyte sizes (Demasi et al., 2021) (Demasi et al., 2021). Additionally, βCD:PMA was synthesized at molar ratio 1:12 mol/mol by electrospinning in fibers with 3 *µ*m diameter. It was applied as micropesticide, loaded with a maximum of 130 mg g^−1^ of *N,N*-diethyl-3-toluamide (DEET), a common insecticide, with a release of 60% in 72 h and 100% at about 350 h (Cecone et al., 2018).

#### Sensors

Cyclodextrin‒materials can be applied as direct or indirect sensor materials via turn‒on or turn‒off of fluorescence both via host‒guest complexation or molecular assembly with fluorescent/luminescent probe molecules.

Fluorescent cyclodextrin‒based probe was obtained via cross-linking of βCD with 4,4′‒diisocyanato‒3,3′‒dimethyl biphenyl (IMP) and tetrakis (4‒hydroxyphenyl)ethane (TPE) as fluorescent ligands. The fluorescent βCD‒polymer was successfully applied for the detection of trinitrophenol and nitrobenzene by turn‒off. These aromatic compounds are used for dye and pesticide synthesis, presenting hazardous effects for human health and possible environment contamination (Danquah et al., 2019). βCD:PMA was prepared at molar ratio of 1:7 mol/mol as fluorescent probe with emission wavelength at 423 nm. The polymeric material has an heterogeneous size distribution with a high polydispersity index. It presents an excellent selectivity for diclofenac (LOD 0.92 *µ*M) in aqueous solution and tablets, whose value is not significantly influenced by the presence of different drugs, probably due to more hydrophobic behaviour of the diclofenac (Nazerdeylami et al., 2021).

#### Catalysts

Being CD nanosponges porous polymeric materials, often with a large active surface area and numerous available active sites, they are considered promising materials to be used as heterogeneous catalysts.

βCD:CDI with 11 m^2^ g^−1^ of active surface and nanoporous structure (5 nm) has promising activity as a metal free catalyst in a one-pot three component condensation reaction (between aromatic aldehydes, methylene compounds and amines) for the synthesis of *N*-containing organic scaffolds (Sabzi and Kiasat, 2018). βCD:EPI (surface area of 11 m^2^ g^−1^ and 5 nm of pore diameter) was applied in a four component solvent-free reaction for the synthesis of spiro [indoline‒3,4′‒pyrano(2,3‒c)pyrazole] and pyranopyrazole derivatives. However, the reaction only proceeds with high yield at 100°C (Jafari Nasab et al., 2018). In another paper, a heteropolyacid was immobilized onto βCD:DPC (3 m^2^ g^−1^ and pore size 14 nm) and applied as catalyst for the synthesis of xanthenes in high yields (87–95%), at reflux, in short reaction times (Sadjadi et al., 2017b). The same authors also described the use of NSs modified with an ionic liquid, that was used in a cascade reaction for the synthesis of benzochromeno-pyrazole derivatives (Sadjadi et al., 2017a). High yields were obtained using solvent-free conditions, at 80°C.

Amino-βCD NSs were covalently bonded to chitosan beads to prepare a thermally stable porous composite, with surface area 11 m^2^ g^−1^. βCD:CS was applied as metal free catalyst for the synthesis of dihydropyrimidinone and octahydroquinazolinone in water, via the Biginelli reaction. The composite reveals excellent catalyst activity, with yields up to 100%, using an ultrasonic assisted procedure at 25°C. The composite was reused and a yield decrease of approximately 10% was observed, after five cycles (Sadjadi and Koohestani, 2021a).

Heterogeneous catalysts based on CD nanosponges and containing embedded inorganic nanoparticles were also described. However, since the aim of this review is focused on pure CDNSs, these studies will not be reported in detail, just a brief classification based on the type of metal used is given: 1) Pd^0^/Pd^2+^ for hydrogenation reactions (Sadjadi and Koohestani, 2021b), cyanation reactions (Khajeh Dangolani et al., 2019), Sonogashira and Heck (Sadjadi et al., 2018b; 2019b); 2) Ag^+^ for redox reactions (Russo et al., 2019); 3) Au for 4‒nitrophenol synthesis (Vasconcelos et al., 2016).

## Future Perspectives

The use of cyclodextrin nanosponges is still at an early stage of wide application in the most diverse areas. Currently, many of the studies described in the literature are based on their application in the pharmaceutical and biomedical areas. However, relevant studies have recently been published on the application of nanosponges in water and, to a lesser extent, soil remediation. However, in this area, it is interesting to note that a 2007 study (Mhlanga et al., 2007) describes the potential of CDNSs for removing chlorinated disinfection by-products (DBPs). Fifteen years later, this issue has become a priority topic for the European Community, so the use of NSs for this purpose should be developed. Concomitantly, other pollutants such as antibiotics, antidepressants and pesticides have molecular structures that suggests a potential high removal efficiency by CDNSs.

The stiffness of cyclodextrins in NSs, due to hyper crosslinking, decreases the degree of freedom of guest molecules that interact with them by supramolecular host-guest interactions. This effect can be used effectively for the development of matrices for Aggregation-Induced Emission (Hong et al., 2009). In general, it is expected that chromophore aggregation leads to a luminescence quenching. However, some molecules have the opposite effect, which is called: Aggregation-Induced Emission. This may result from the restriction of intramolecular rotation (RIR). CDNSs seem to be potential matrices to promote the RIR mechanism and, thus, lead to luminescence emission by the chromophores. The impact of this phenomenon might be relevant in areas such as optoelectronics and sensors.

Using a similar approach, we think that nanosponges could play an important role in the development of sensors with very low detection limits. The preconcentration of pollutants is often a necessary condition for their detection. NSs could have a similar effect through their use in monoliths or by using membranes. In the latter case, we envisage the use of, e.g., cellulose acetate or PVA as matrices for incorporation of CDNSs.

We also believe that the use of NSs for heterogeneous catalysis is incipient. Most of the conventional asymmetric synthesis methods involve the use of toxic organic solvents, chiral metal complexes and homogeneous catalysis methods, which makes them unsustainable. On the other hand, usage of heterogeneous matrices with high density of hydrophobic cavities capable of enantiomeric discrimination could be a valuable alternative. CDNSs can accommodate hydrophobic molecules inside and interact efficiently with aqueous media in the exterior, opening the way to the development of greener asymmetric chemical reactions. Additionally, CDNSs can also be modified with metallic nanoparticles, allowing the development of more sustainable organometallic heterogeneous catalysts.

It should be mentioned that some of these suggestions and views have been tested by authors and preliminary results are available. However, we strong believe that CDNSs either alone or in the presence of other polymers still have a promising future not only for fundamental interest but especially for a wide range of not yet unveiled applications.

## Glossary

4C3MP: 4‒chloro‒3‒methylphenol
2,4CP: 2,4‒dichlorophenol
2CP: 2‒chlorophenol
2,4,6CP: 2,4,6‒trichlorophenol
2,4D: 2,4-dichlorophenoxy acid
2-HEDS: 2-hydroxyethyl disulfide
2NP: 2‒nitrophenol
4NP: 4‒nitrophenol
2,4NP: 2,4‒dinitrophenol
4nNP: 4‒n‒nonylphenol
4nOP: 4‒n‒octylphenol
4tOP: 4‒tert‒octyphenol
AC: activated carbon
am6: 1,6-hexanediamine
am12: 1,12-dodecanediamine
AMD: 1‒(3‒dimethylaminopropyl)‒3‒ethylcarbodiimide hydrochloride
BDE: 1,4-butanediol diglycidylether
BPA: Bisphenol A
BPAF: Bisphenol AF
BPF: Bisphenol F
BPS: bisphenol S
CA: citric acid
CDs: cyclodextrins
CDI: 1,1′-carbonyl diimidazole
CDNSs: cyclodextrin nanosponges
CHI: dicyclohexylmethane-4,4′-diisocyanate
CHS: cholesterol hydrogen succinate
CMP: 4,4′-bis(chloromethyl)-1,1′-biphenyl
CPA: 4‒chlorophenoxyacetic acid
CyCa NSs: CD-calixarene NSs
CR: congo red
CTS: chitosan
CV: cristal violet
DABCO: 1,4-diazabicyclo(2,2,2)octane
DBPs: chlorinated disinfection by-products
DEET: N,N‒diethyl‒3‒toluamide
DMC: dimethyl carbonate
DPC: diphenyl carbonate
EDTA: ethylenediaminetetraacetic acid dianhydride
EE: encapsulation efficiency
EPI: epichlorohydrin
Et3N: triethylamine
FPS: 4,4′‒difluorodiphenylsulfone
GSH-NS: glutathione-responsive NSs
HDI: 1,6-hexamethylene diisocyanate
IMP: 4,4′‒diisocyanato‒3,3′‒dimethyl biphenyl
MB: methylene blue
MBA: N,N′-methylene bisacrylamide
MDI: methylene diphenyl diisocyanate
MIPs: molecularly imprinted polymers
MO: methyl orange
MPP: 2,2′‒azobis(2‒methylpropionitrile)
NADES: deep natural eutectic solvents
NDCA: 2,6-naphthalene dicarboxylic acid
NIP-NSs: non-molecularly imprinted polymers
NSs: nanosponges
OXY: oxyresveratrol
P5: pillar(5)arene
PAA: polyamidoamine
PD: 4,4′-bipyridine
PDI: polydispersity index
PDS: 1,4‒phenylene diisocyanate
PFP: pentafluoropyridine
PMA: pyromellitic dianhydride
RB: rhodamine B
RIR: restriction of intramolecular rotation
SBET: surface area
SFsafranin O; TA: safranin O; TAtannic acid
TCP: 2,3,4,6‒tetrachlorophenol
TDI: oluene 2,4-diisocyanate
TFP: tetrafluoroterephtalonitrile
THTS: 5,5′,6,6′‒tetrahydroxy-3,3,3′,3′-tetramethylspirobisindande
TPC: terephthaloyl chloride
TPE: tetrakis(4‒hydroxyphenyl)ethane
TTI: triphenylmethane‒4,4′,4″‒trisocyanate
